# Single Electrode Deep Brain Stimulation with Dual Targeting at Dual Frequency for the Treatment of Chronic Pain: A Case Series and Review of the Literature

**DOI:** 10.3390/brainsci7010009

**Published:** 2017-01-13

**Authors:** Milo Hollingworth, Hugh P. Sims-Williams, Anthony E. Pickering, Neil Barua, Nikunj K. Patel

**Affiliations:** 1 Department of Neurosurgery, North Bristol Trust, Bristol BS10 5NB, UK; milo.hollingworth@gmail.com (M.H.), neil.barua@nbt.nhs.uk (N.B.); 2 Department of Neurosurgery, Sheffield Teaching Hospital, Sheffield S10 2JF, UK; simswilliams@doctors.org.uk; 3 School of Physiology, Pharmacology & Neuroscience, University of Bristol, Bristol BS8 1TD, UK; tony.pickering@bristol.ac.uk

**Keywords:** deep brain stimulation, CMPf, PAG/PVG, pain, pain pathways

## Abstract

Deep Brain Stimulation (DBS) has been used to target many deep brain structures for the treatment of chronic pain. The periaqueductal grey and periventricular grey (PAG/PVG) is an effective target but results are variable, sometimes short-lived or subject to tolerance. The centromedian intra-laminar parafascicular complex (CMPf) modulates medial pain pathways and CMPf DBS may address the affective aspects of pain perception. Stimulation of multiple deep brain targets may offer a strategy to optimize management of patients with complex pain symptomatology. However, previous attempts to stimulate multiple targets requires multiple trajectories and considerable expense. Using a single electrode to stimulate multiple targets would help overcome these challenges. A pre-requisite of such a technique is the ability to use different stimulation parameters at different contacts simultaneously on the same electrode. We describe a novel technique in 3 patients with chronic pain syndromes for whom conventional medical and/or neuromodulation therapy had failed using a single electrode technique to stimulate PVG/PAG and CMPf at dual frequencies.

## 1. Introduction

### 1.1. Deep Brain Targets for the Treatment of Pain

For over 60 years deep brain stimulation (DBS) has demonstrated significant analgesic benefits. James Olds and Peter Milner found stimulation of septal regions in rodents elicited self-stimulation overwriting normal survival behaviours [1]. In attempts to treat patients with schizophrenia, stimulation of septal structures yielded serendipitous pain relief [2]. Sufferers of malignant oncological diseases and rheumatoid arthritis provided a willing and ethically justifiable cohort and, indeed, septal region DBS proved moderately effective in early studies. However, due to variable and non-sustained responses, finding alternative deep brain targets became a priority. Consequently targets range from the internal capsule (IC) [3] to thalamic structures such as the somatosensory thalamus, centromedian intralaminar parafascicular complex (CMPf) [4], to the periventricular and periacqueductal grey (PVG/PAG) [5], to the nucleus accumbens (NAc) [6] and anterior cingulate cortex (AC) [7]. However, despite this array of deep brain targets, the complexity of pain management is a persistent challenge demanding new approaches. We provide a technical note of a novel technique and a review of the literature. Stimulation of PVG/PAG and CMPf is the focus of this review and technical note and will be described separately. The rationale and effects of targeting the other deep brain nuclei are important to place the treatment of chronic pain by DBS in context.

### 1.2. Anterior Cingulate

The first published case of AC DBS was reported by Spooner et al. [8] in a patient with neuropathic pain secondary to spinal cord injury. He received bilateral and unilateral DBS to the AC and PVG respectively. Pain relief was assessed using the Visual Analogue Scale for pain (VAS) and by tracking pain medication usage. Bilateral cingulate stimulation resulted in improved affect and was associated with improved subjective analgesic properties relative to PVG stimulation alone. Boccard et al., in 2014 [7] presented a case series of AC stimulation in 15 patients with chronic pain with a range of aetiologies including failed back surgery syndrome, poststroke pain, brachial plexus injury, cervical spinal cord injury, head injury, and pain of unknown origin. Using several pre- and post-operative pain measures they detected statistically significant overall improvement in reported pain. In 5 patients VAS decreased to less than 4 on a scale of 1 (no pain)–10 (very severe pain). No major adverse events were reported. Although, this study is limited by its heterogenous population and assessment measures that under emphasize the affective components of pain, this study demonstrates that AC stimulation is a useful option. As such, preclinical and clinical studies have explored the importance of the AC in pain perception, which has been subject to comprehensive review [9].

### 1.3. Nucleus Accumbens

NAc, forms an extension of the ventral striatum, which is involved in reward processing. DBS of the NAc is used in the treatment of depression and obsessive-compulsive disorder [10,11]. NAc also projects inhibitory projections to the medial thalamus [12] and thereafter to dorsal horn neurons to modulate pain perception [13]. The NAc together with the prefrontal cortex, insula and AC mediates the affective component of pain [12,13]. One case of post-stoke pain has been treated using DBS targeting the NAc and PVG simultaneously to great effect [6]. In our case of post-stroke pain described herein, stimulation of the NAc was combined with dual targeting of the PVG/PAG, however this was not found to be helpful.

### 1.4. The Somatosensory Thalamus

Somatosensory thalamus consisting of the ventroposterior lateral (VPL) and medial (VPM) nuclei have been targeted to treat chronic pain for over 40 years [14,15] Hosobuchi et al., in 1973 treated five patients with facial pain secondary to retrogasserian rhizotomy. Chronic stimulation of the contralateral VPM induced a paresthesia, which improved pain tolerance in 4 out of 5 patients [14]. The explanation for targeting the somatosensory thalamus is its aberrant neuronal firing observed in chronic pain [16] presumably driven by the absence of normal sensory input [17]. Melzack and Wall support this idea in their gate control theory [18], postulating that low threshold somatosensory pathways inhibit pain perception. Stimulation of this pathway was expected to reduce neuropathic pain. This has been supported in animal models where VPL stimulation inhibited spinothalamic nociceptive neurons [16,19]. Indeed, in a series of 12 patients with neuropathic pain secondary to brachial plexus injuries and phantom limb pain, 11 demonstrated improvement in pain scores following stimulation of VPL [20].

### 1.5. PVG/PAG

**Figure 1:**
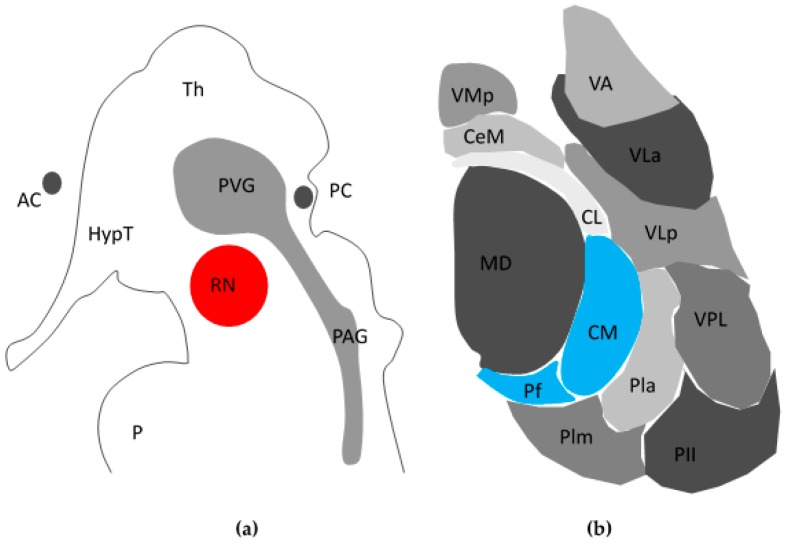
Schematic view of peri-aqueductal grey/Periventricular grey (PAG/PVG) and centromedian intralaminar parafasciular complex (CMPf). (**a**) Sagittal cross-sectional schema of PVG/PAG within midbrain/diencephalon; (**b**) Axial cross section of left thalamus demonstrating CMPf (**blue**) adapted from Weigel and Kraus, 2004 [30]. AC: Anterior Commissure; HypT: Hypothalamus; RN: Red Nucleus; PC: Posterior Commissure; Th: Thalamus; P: Pons; MD: Mediodorsal nucleus; VMp: Ventral Posteromedial nucleus; CeM: Central Medial nucleus; CL: Centrolateral; Plm: Medial nucleus of Pulvinar; Pll: Lateral nucleus of Pulvinar; Pla: Anterior nucleus of Pulvinar; VPL: Ventral Posterior Lateral nucleus; VLp: Ventrolateral Posterior nucleus; VLa: Ventrolateral Anterior nucleus; VA: Ventral Anterior nucleus.

The PVG/PAG (see Figure 1a) is the most promising target in DBS for chronic pain [21]. The first descriptions of PVG/PAG DBS in humans demonstrated relief of somatoform and nociceptive pain in both the acute and chronic settings [22,23]. This was consistent with descriptions of a PAG- derived descending inhibitory system modulating nociceptive inputs at a spinal level [24]. Indeed, recent evidence demonstrates PAG DBS causes a focal reduction of opioid binding in areas of electrostimulation consistent with the release of endogenous opioid peptides [25]. This is in keeping with several studies implicating opioids in PAG/PVG mediated attenuation of nociception. The analgesic effect of PAG DBS in both animal models and humans is reversible with the opioid antagonist naloxone [26] and opioid receptor binding density is also remarkably high [27]. However, the picture is complicated by blinded studies of DBS PAG patients whose analgesia was not fully reversed by naloxone and similarly failed to show cross-tolerance to systemically administered opioids [5]. Such findings raise the possibility of non-opioidergic mechanisms of PAG analgesia [28,29] painting a more complex picture of pain control. Indeed, although stimulation of the PAG/PVG provides long-term relief in 79% of patients with nociceptive pain, it is less effective in central and de-afferentation pain syndromes [28]. Taken together although PAG/PVG stimulation are an important focus, its mechanism of action is complex and innovation is still required to design better treatments for patients. 

### 1.6. Centromedian Parafasciculus Complex (CMPf)

Intra-operative stimulation of the CMPf and the intra-laminar zone achieves variable results [30,31], which may explain why it has received less attention compared to other targets. The CMPf (Figure 1b) was first described by Jules Bernard Luys [32], a 19th century Neurologist who also was first to describe the subthalamic nucleus. Dividing the medial and lateral thalamus, the internal medullary lamina contains anterior nuclei and posterior nuclei, the latter of which contains the CMPf [31]. The CMPf has afferents from the ventral posterolateral thalamus (VPL), spinothalamic tract (STT) and trigeminal lemniscus, and efferents to the striatum, cortex, and AC. It is responsive to noxious stimuli in rodents, large animals and primates [30]. However, neurones in the CMPf do not respond in a binary fashion but differentiate stimulus intensity. Firing of the VPL to nociceptive inputs carried within the STT actually inhibit activity in the CMPf, giving rise to a “thalamic gate theory” akin to Melzack and Wall’s ideas of pain modulation in the spinal cord [19]. Inputs to the striatum and the anterior cingulate also suggest the CMPf may modulate affective and behavioural responses suggesting that the CMPf is central to the concept of the medial pain pathway [33].

In human studies, the CMPf demonstrates increased background activity in neuropathic pain [34]. Similarly, the CMPf also expresses a high density of opiate receptors in the rested state [35,36]. Rinaldi et al. found that the intra-laminar thalamic nuclei, including the CMPf are also active in de-afferentation pain [37] and moreover, stimulation of the PVG reduces CMPf firing suggesting critical connectivity of the CMPf to pain-encoding structures [38]. Hariz and Bergenheim found that CMPf stimulation or ablation was helpful in the treatment of central pain and de-afferentation pain [39]. However in a prospective study of bilateral thalamic stimulation, CMPf stimulation only provided short-term relief from neuropathic pain [40]. Despite this it remains a promising target due to its anatomical and electrophysiological profile.

### 1.7. Dual Stimulation

The interconnectivity within the central nervous system prompts the consideration of stimulating multiple brain structures simultaneously to recruit complex neuronal processes involved in pain sensation and perception. In a meta-analysis the most successful technique to provide long term analgesia in 87% of cases reported was stimulation of both the PVG/PAG and Sensory thalamus/IC [21]. In the same way, stimulation of PVG and NAc have been used to treat post-stroke pain successfully [6]. 

We have previously published outcome data (*n* = 3) for dual stimulation of PAG/PVG and CMPf in the treatment of trigeminal anaesthesia dolorosa (TAD) [41]. The mean VAS was acutely reduced from 55 mm to 24 mm for PAG stimulation and from 67 mm to 22 mm for CMPf stimulation. PAG/PVG and CMPf stimulation were associated with pleasant warmth and improved pain tolerance respectively. Dual stimulation elicited both these features and was ultimately associated with reduction in analgesia requirements. This study by our group [41] and those reviewed by Bittar et al., [21] demonstrate the non-antagonistic effects of dual stimulation in the treatment of chronic pain. However, dual stimulation has practical considerations. For example, in our case series of 3 patients receiving dual stimulation for TAD, all patients required separate generators and trajectories per target to deliver dual stimulation parameters [41]. This is in itself multiplies the risk of haemorrhagic and infective complications as well as the financial cost. However, whilst treating a patient with phantom limb pain (not included in this series), stimulation of the proximal contacts of a PAG/PVG-targeted electrode yielded surprisingly favourable results. Closer inspection of these proximal contacts demonstrated their location within the CMPf lying along the trajectory directed toward the PAG/PVG (Personal Communication N.P.). The ability to target both the CMPf and PAG/PVG simultaneously along a single trajectory provided a solution to the increased cost and risk of surgical morbidity associated with dual stimulation by “striking two birds with one stone”.

Single electrode dual targeting offers a practical solution to a risky and expensive problem and may encourage others to further explore the potential of dual stimulation. The ultimate aim would be to exploit different pain circuitry to provide synergistic modulation of complex pain symptomatology. However, such synergism can only be achieved by stimulating targets at their optimal parameters. Hence, single electrode dual targeting techniques require generators equipped to deliver different frequencies simultaneously. Through collaboration with Boston Scientific^®^ (Marlborough, MA, USA), we exploited the versatility of the Boston Scientific Vercise^TM^ generator (Boston Scientific Inc, Marlborough, MA, USA) to perform single electrode dual target dual stimulation PVG/PAG and CMPf DBS in 3 cases with chronic pain who either failed or were not amenable to conventional spinal cord stimulation.

## 2. The Technique

**Figure 2:**
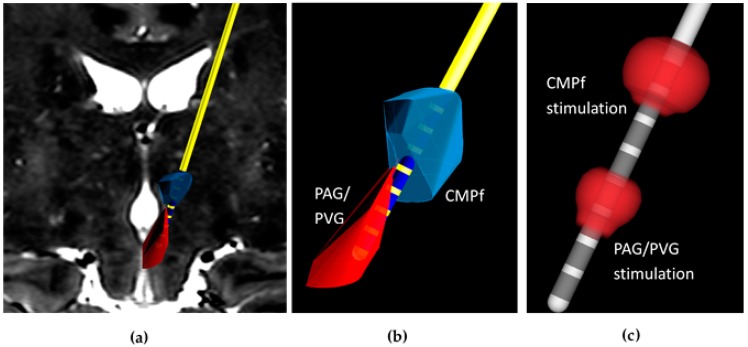
Dual frequency stimulation using a single electrode technique to target the Periaqueductal Grey/Periventricular Grey (PAG/PVG) and Centromedian Intralaminar Parafasciular complex (CMPf) in the treatment of chronic pain. (**a**) Tracings of the CMPf (**blue**) and PAG/PVG (**red**) in coronal plane undergo volumetric reconstruction using NeuroInspire^TM^ software to create 3-dimensional structures for robot-guided DBS electrode implantation; (**b**) Contacts 1–3 and 5–8 are embedded within the PAG/PVG and CMPf respectively along the same trajectory; (**c**) Spherical electrical fields (**red**) at contacts 4 and 8 stimulate the PAG/PVG and CMPf respectively yielding analgesia in a case of refractory phantom limb pain.

The technique previously developed by this group is based on magnetic resonance imaging (MRI) directed electrode implantation using implantable guide tubes [42]. Pre-operative planning scans including T1 and T2 MRI and Computer Tomography Angiogram (CTA) are co-registered on in-house adapted Neuroinspire^TM^ software (Renishaw PLC., Wotton-under-Edge, UK). Deep brain trajectories to the CMPf and PAG are planned with proximal electrode contacts positioned within CMPf and distal contacts within PAG/PVG (see Figure 2a,b). Simultaneous targeting of both structures with a single trajectory requires the use of a DBS system with contacts spanning a sufficient distance and with the facility to use different stimulation parameters at each target. We have therefore opted to use the Boston Scientific Vercise^TM^ system to achieve these aims.

In the operating theatre, the patient is placed under general anaesthetic and a Leksell frame fitted. The patient is then placed in the head-holder of the Neuromate stereotactic robot (Renishaw PLC., Wotton-under-Edge, UK). Using intra-operative post-contrast three-dimensional fluoroscopy (O-Arm, Medtronic, Minneapolis, MN, USA), the patient position is registered and stereotactic co-ordinates outputted to the robot. A linear scalp incision is made and the periosteum retracted. The robot is driven to the first position on the skull and a multi-featured burr hole drilled. Using custom-made tooling a track is dissected using 1.3 mm and 1.7 mm outer diameter (OD) guide rods which traverse both CmPf and PAG. Prior to implantation of the electrode, a radio-opaque carbothane guide tube and stylet (Renishaw PLC, Wooton-under-Edge, UK) are inserted to target. The guide tube is cut 20 mm shorter than the stylet in order to ensure exposure of the electrode contacts within the deep brain targets. Insertion of guide tubes and stylets are visualized without the metal artifact and targeting accuracy verified by performing O-arm imaging after electrode insertion. 

Once targeting accuracy is confirmed by co-registering the on-table imaging with the pre-operative plan, the stylet is removed and replaced with a Boston Scientific Vercise DBS system (model DB2201). Integration of the Boston Vercise^TM^ DBS with Neuroinspire^TM^ software (Renishaw PLC, Wooton-under-Edge, UK) allows visualization of electrode contacts within the target structures to 0.3 mm accuracy (Figure 2a,b).

Through personal collaboration between this group and Boston Scientific^®^, Boston Scientific Vercise^TM^, model DB1110 generator was optimised for dual frequencies output (each ranging from 2 to 225 Hz) at two separate locations along the electrode (Figure 2c, Table 1). Our robot-guided technique co-registered with pre-and perioperative imaging allows precise knowledge of contact location so contacts within the PAG/PVG and CMPf can be selectively activated. We performed this procedure in 3 patients with different aetiologies of intractable chronic pain.

### 2.1. Case 1

52-year old scientist with a 2 year history of progressive left-sided facial pain secondary to Lyme’s Disease contracted during field work was reviewed by neurosurgery following extensive contact with pain services. Her symptoms consisted of complete hypoesthesia to the left face with associated paraesthesia and allodynia. Supramaximal dosing of gabapentin at 5.4 g daily reduced her symptoms but was associated with significant cognitive disturbance and sedation. Pulsed radiofrequency therapy of the trigeminal ganglion failed to achieve benefit. The patient suffered persistent paraesthesia and irritation interfering with her activities of daily living. The patient was admitted for single electrode dual target surgery, which was performed under general anaesthetic with robot assistance as described above [27]. Implantation of a guide tube via a right transfrontal approach allowed delivery of an electrode to 96.6 mm passing through the right CMPF terminating in the right PAG. Stimulation of the CMPf and PAG/PVG resulted in a pleasant facial paresthesia and cold sensation respectively, dual stimulation subjectively provided preferable analgesic effects. Two days following surgery the generator was activated using dual frequencies (Table 1). At 3 years post-surgery, the patient only suffered minimal symptoms with full return to her activities using concomitant DBS stimulation and gabapentin therapy weaned to 1.8 g daily (Table 1).

### 2.2. Case 2

**Figure 3:**
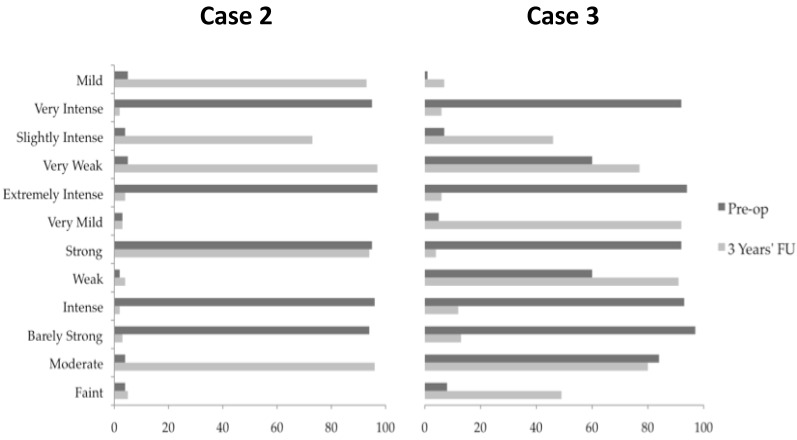
Pre-operative and post-operative Descriptor Differential Scale (DDS) [43] for Cases 2 and 3. Pre-operative and post-operative DDS measurements were performed for Case 2 and Case 3. Patients were asked to mark along each line the extent to which the description applies to their symptoms between 0 and 100. Single electrode dual target dual stimulation at 3 years follow-up decreased the severity of pain consistently in patients 2 and 3 compared to pre-operative assessments. Post-operative DDS measurements were not available for Case 1.

A 35-year old woman with left arm phantom pain was treated with DBS. Her pain developed after a severe episode of self-harm 10 years prior. Her tendons and nerves were cut at the proximal forearm resulting in contractures and de-afferentation pain. Above elbow amputation was eventually required; however, this resulted in worsening pain. Despite successful neuroma excision, a prosthesis was not tolerated. After input from a specialist pain team and multiple pharmacological trials, the patient underwent a trial of spinal cord (dorsal column) stimulation, which was ineffective. Her predominant complaint was cold pain centered over her phantom elbow requiring 2.7 g Gabapentin, 400 mg Tramadol, Baclofen 20 mg, and Amytriptyline 150 mg daily for pain control. To control her symptoms, the patient had become reliant on a hot compress applied to her stump fixed in place with a bandage. Keeping the compress hot had become a pre-occupation leading to significant limitation of her outdoor activities, even making attendance of her hospital follow-up problematic. Following thorough psychiatric assessment, the patient was admitted for single electrode dual target surgery as described. Via a right transfrontal approach a guide-tube was implanted and an electrode passed along the trajectory to a depth of 96.5 mm passing through the CMPf and PAG/PVG. Following activation of the generator (Table 1) the patient’s predominant cold pain was reduced. PAG/PVG stimulation alone achieved a warm glowing sensation throughout the phantom arm, hand and fingers, leaving only a cool sensation in her finger nails. The addition of CMPf stimulation resulted in reconfiguration of the phantom limb leading to dissipation of her pain at the elbow (Figure 3, Table 1). The elbow pain was however later replaced by a less severe pain in her phantom wrist (5/10 severity). It was not possible to control the new wrist pain without a return of the elbow pain. At 3 years follow-up, the patient was no longer reliant on her hot compress, which allowed her to leave her home and return to independent living.

### 2.3. Case 3

A 49-year old man presented with an 8-year history of repeated strokes. At 41 years of age he suffered a right middle cerebral artery infarct resulting in contralateral hemiparesis. At the time of his infarct, the patient reported pain in the left upper limb, neck and leg with sparing of the face, which failed to abate following his recovery. The pain was described as burning/pulling sensation that was present all the time and it was associated with hypoesthesia. The patient was diagnosed with central pain syndrome and received extensive input from pain physicians over the intervening eight years with little alleviation in his symptoms. He was referred for consideration of DBS to treat his chronic pain. He underwent single electrode right CMPf and PAG/PVG targeting combined with targeting of his right NAc via a separate trajectory. NAc stimulation was found to be unhelpful and its stimulation was quickly terminated. Stimulation of the CMPf and PAG/PVG separately resulted in a warm paresthesia and reduced allodynia respectively. The patient continued dual CMPf PAG/PVG stimulation with improvement in his pain severity at 3 years follow-up (Figure 3, Table 1).

### 2.4. Testing and Optimization of Dual Frequency Dual Stimulation

Testing was performed at 2.5-min intervals during each assessment. Baseline pain was established prior to the next target being stimulated. Each patient had both pulse generators switched off prior to testing. Pain was allowed to stabilize, which took up to 60 min. CMPf monostimulation was performed and descriptions of pain were recorded. The pulse generator was then switched off to allow pain to re-stabilise. The PVG/PAG was then stimulated singly; the process was repeated before finally assessing dual stimulation. Extensive description of this method is previously described by our group [41].

## 3. Discussion

Dual target stimulation with a single electrode represents a natural progression from dual target stimulation with multiple electrodes. This strategy requires the use of DBS technology with the flexibility to deliver different stimulation parameters at the proximal and distal contacts of an electrode. Such requirements may necessitate the use of multiple generators or a specially adapted generator, such as the Boston Scientific Versize^TM^ DBS system, to deliver more than one set of stimulation parameters simultaneously.

This technique may be a potential solution to a complex condition such as intractable pain where even recent advances in neuromodulation, such as spinal cord stimulation and intrathecal therapy, have failed. Dual stimulation of the PVG/PAG and CMPf could help to modulate different symptom components including affective and nociceptive features in order to optimise long-term response and reduce tolerance. Dual targeting with one trajectory can also offer improved safety and cost-effectiveness by minimising the number of electrode insertions, use of operating time and resources. In this article, we describe 3 cases of robot-guided single electrode DBS to stimulate the CMPf and PAG/PVG and the methodological steps required to provide adequate accuracy, safety and efficacy. This further establishes the combined roles of CMPf and PAG/PVG in the control of chronic pain particularly those with de-afferentation and central pain syndromes. This also may allow us to improve the care of patients with increasingly complex pain conditions, and perhaps even neurological conditions beyond the current horizons of “standard” functional neurosurgery. Treatments for epilepsy, disordered consciousness, psychiatric illness and neurodegenerative diseases could potentially all be augmented using such a technique. Already, dual targeting has been explored outside the realms of chronic pain by way of simultaneous stimulation of globus pallidus internus (GPi) and externus stimulation in Parkinson’s disease [44], GPi and subthalamic nucleus in Huntington’s disease [45] and ventral intermediate nucleus and the subthalamic area in Holme’s tremor [46]. Indeed, many promising applications of DBS such as Tourette’s syndrome [47], epilepsy [48] and minimally conscious states [49] boast a selection of possible deep brain targets. Single electrode dual target dual stimulation DBS, either unilaterally or bilaterally, could be used to exploit two targets simultaneously and, with necessary developments in electrode and generator design, possibly more. Such developments could help explore conditions more ethically and more comprehensively allowing patients multiple therapeutic options, whilst providing opportunities to compare the efficacy of stimulating different structures without the need for further operations. In our case series, we describe a novel concept overcoming the safety and financial implications of multiple targeting in DBS. This technique will hopefully encourage others to consider its potential in chronic pain and other neurological diseases in both pre-clinical and clinical studies. Single electrode dual target dual stimulation DBS may also facilitate further research previously restricted by single target DBS, as dual targeting can be used to compare the stimulation effects of different brain targets without increasing surgical morbidity or multiplying costs.

## 4. Limitations

DBS for chronic pain remains a challenging area for both patient and physician. Accumulative evidence from hundreds of patients is available; however, it is observational in nature and derived from cohort studies, case-series and reports describing heterogeneous groups of patients, using various stimulation and neuroimaging technologies to target different deep brain structures. This case series does little to clarify the best way to treat chronic pain, but as a technological development it raises the possibility of treating chronic pain, and maybe other neurological diseases, in a new way. We cannot make assertions regarding the efficacy of our therapy versus other treatment options owing to the small number of patients, the absence of a control group and the subjectivity of our recorded outcomes. Our series would be improved by being able to demonstrate assessments of affective, as well as nociceptive, aspects of pain before and after surgery in all patients. However, this was not possible. We can show that our technique was safe and well tolerated. It also provided preferable analgesic relief for three patients who failed to benefit from conventional approaches.

## Figures and Tables

**Table 1:** Stimulation parameters and overall Visual Analogue Scales (VAS) at pre-operative assessment and 3 years’ follow-up (FU) in single electrode dual targeting of the Periaqueductal grey/Periventricular grey (PAG/PVG) and Centromedian Intralaminar Parafasciular complex (CMPf) for three patients with chronic pain syndromes.

	Diagnosis	PAG/PVG Stimulation			CMPf Stimulation			Overall VAS (mm)		Medication, Total Daily Dose	
		mA	µs	Hz	mA	µs	Hz	Pre-op	3 Years’ FU	Pre-op	3 Years’ FU
Case 1	Trigeminal anaethetica dolorsa	4.5	60	10	4.5	60	128	44	5	Gabapentin 4800 mg	Gabapentin 1800 mg
Case 2	Phantom limb pain	4.0	90	10	4.0	90	132	94	56	Tramadol 400 mg, Gabapentin 2700 mg, Temazapam 10 mg, Amitriptyline 150 mg	Mirtazepine 30 mg Tramadol 400 mg Baclofen 40 mg, Temazapam 10 mg, Amitriptyline 150 mg
Case 3	Post-stroke Pain	3.5	110	10	4	90	132	98	32	Carbamazepine 800 mg, Duloxetine 120 mg, Pregabalin 450 mg	Carbamazepine 800 mg, Duloxetine 120 mg, Pregabalin 450 mg
